# Media Exposure and Its Association With Vaccine Attitudes, Intentions, and Hesitancy: Systematic Review

**DOI:** 10.2196/74280

**Published:** 2026-04-28

**Authors:** Francesco Leonforte, Vito Nicosia, Paola Comite, Giustino Morlino, Antonio Mistretta

**Affiliations:** 1 Department of Integrated Hygiene, Organizational, and Service Activities (Structural Department) Health Management University Hospital Polyclinic “G. Rodolico—San Marco” Catania Italy; 2 Department of Medical, Surgical Sciences and Advanced Technologies “G.F. Ingrassia” University of Catania Catania, CT Italy; 3 Accreditation and supply network Area Local Health Authority “Roma 1” Rome Italy; 4 Scientific Communication Service National Institute of Public Health (Istituto Superiore di Sanità) Rome Italy

**Keywords:** vaccine literacy, vaccine hesitancy, vaccine adherence, communication, traditional media, digital media, sociodemographic factors, systematic review

## Abstract

**Background:**

Vaccine hesitancy, amplified by the COVID-19 “infodemic,” has emerged as a pressing public health challenge. Communication strategies are pivotal for enhancing vaccine literacy, countering misinformation, and sustaining immunization programs.

**Objective:**

This systematic review evaluates the association between communication channels and vaccine hesitancy and adherence, while examining the moderating role of sociodemographic factors.

**Methods:**

A PRISMA (Preferred Reporting Items for Systematic Reviews and Meta-Analyses)–compliant search was conducted across PubMed, Scopus, and Web of Science, yielding 17,407 records screened according to predefined eligibility criteria (peer-reviewed studies with N>1000 adults assessing communication media targeting vaccine hesitancy and adherence, excluding pediatric, health care–specific, and non-English research). After full-text assessment, studies were appraised using the Modified Medical Education Research Study Quality Instrument for methodological quality and the Joanna Briggs Institute Critical Appraisal Checklist, ROBIN-I (Risk of Bias in Nonrandomized Studies of Interventions), or RoB 2.0 (Risk-of-Bias 2.0 tool for randomized trials) tools for risk of bias.

**Results:**

Thirty-six studies were included (26 cross-sectional, 4 quasi-experimental, 4 randomized controlled trials, 1 cohort, and 1 global analysis). Randomized and nonrandomized experimental studies demonstrated that tailored communication strategies delivered via radio, web platforms, and social media significantly improved vaccine acceptance. Adaptive public health campaigns achieved up to an 8% weekly increase in uptake in Madagascar (relative risk 1.08; 95% CI 1.01-1.15) and a 7.8% higher vaccination rate among Nigerian adults at first follow-up compared with controls. Digital and social media campaigns effectively reduced hesitancy and enhanced trust among hesitant pregnant women in the United States. Sociodemographic factors significantly moderated communication outcomes: a COVID-19 chatbot proved most effective among individuals with lower education and minority backgrounds, while religiosity (b=0.17; 95% CI 0.05-0.30; t810=2.80; P=.005) and cultural congruence (odds ratio 1.89; P<.01) influenced message credibility and engagement, respectively. The persuasive effect of online memes on COVID-19 vaccine intentions was not significantly influenced by gender (P=.83), age (P=.60), or political orientation (P=.44). Age-specific effects were observed, with greater responsiveness to a social media campaign among adults aged 25-34 years and reduced hesitancy among older groups. Multiple cross-sectional studies indicated higher adherence among audiences exposed to traditional media (television, radio, newspapers) and lower trust among social media users. Other studies suggested significant influences of gender, age, socioeconomic status, education level, and political orientation.

**Conclusions:**

By synthesizing fragmented evidence, this review provides a systematic examination of the interplay between multichannel media and vaccine acceptance. It diverges from existing literature by integrating both traditional and digital media perspectives through the lens of sociodemographic moderation. This work offers a critical framework for public health interventions, advocating for rigorous longitudinal research to establish definitive causal links between communication and behavior. Consequently, these findings support a “precision” communication model, enabling the development of culturally congruent strategies tailored to specific recipient profiles to bolster vaccine adherence.

**Trial Registration:**

PROSPERO CRD42025637441; https://tinyurl.com/4r9w83kw

## Introduction

Communication in the context of vaccination is a global priority, essential to the success of vaccination campaigns and the safeguarding of public health [1]. Although vaccines are a key tool for preventing infectious diseases, vaccine hesitancy continues to undermine coverage and expose vulnerable populations to risk [2,3]. The COVID-19 pandemic has exacerbated these issues, highlighting the importance of targeted, effective communication strategies to rebuild trust in vaccines and promote acceptance [4].

The World Health Organization (WHO) defines vaccine hesitancy as a delay in acceptance or refusal of vaccination despite availability, driven by distrust, safety concerns, misinformation, and sociocultural influences [5]. By contrast, vaccine literacy is the ability to acquire, understand, and apply vaccine-related information; it is strengthened by transparent, empathetic, and participatory communication and is particularly effective for individuals who are uncertain or misinformed [6].

The COVID-19 “infodemic” has exemplified the massive spread of unverified claims, conspiracy theories, and misleading digital information that spreads faster than scientific evidence [7].

In this context, communication channels play a decisive role. It is possible to distinguish between traditional media (television, radio, and print) and digital/social media (internet-based platforms such as Facebook, Twitter, and Instagram). These channels differ substantially in communication dynamics, speed, audience targeting, and perceived credibility [8]. Traditional media generally operate through a 1-way flow of information, in which professional gatekeepers deliver content to a broad audience. Television and radio are used for public service announcements, informative spots, and educational programs, and are particularly useful in communities with limited internet access [9,10]. Print media, such as newspapers and magazines, offer analyses and contextualization of complex data, providing scientific and technical insights [11,12]. Social and digital media enable 2-way, interactive exchanges and promote user-generated content, offering immediacy and flexibility that are otherwise hindered by production and editorial constraints in traditional outlets [13,14]. These platforms also allow for highly targeted engagement and real-time monitoring of public sentiment, leveraging emotional appeals and establishing a personal connection with the audience [15]. However, they are also more vulnerable to misinformation and echo chambers. Although traditional media are often perceived as more credible and reliable, they can sometimes present negative or inaccurate messages, thereby increasing confusion and uncertainty among the public [16,17].

Despite their differences, the 2 systems are increasingly interdependent. Social platforms frequently amplify the reach of traditional outlets by directing audience attention, while traditional media often incorporate social media trends into their programming. This mutual influence has given rise to a hybrid, integrated, and more diversified media environment [18]. Vaccine communication is a widely debated topic in recent literature [19]. Numerous academic works have detailed the impact of various strategies aimed at enhancing vaccine literacy and counteracting hesitancy [20-23], with particular attention to sociodemographic variables [24,25]. Despite growing evidence, it remains crucial to address the lack of systematic reviews in this field.

This systematic review, therefore, aims to provide a deeper understanding of the role of communication means in promoting vaccine literacy and countering vaccine hesitancy. The specific objectives are as follows:

- Evaluate and compare the correlation between communication means and vaccine attitudes, hesitancy, and adherence.
- Investigate the role of sociodemographic factors.
- Identify gaps in the literature and future directions.

## Methods

### Overview

This systematic review was conducted in accordance with the PRISMA (Preferred Reporting Items for Systematic Reviews and Meta-Analyses) guidelines. The completed PRISMA checklist is provided in Multimedia Appendix 1, ensuring transparency and reproducibility of the results [26]. The screening process was conducted in a double-blind manner in pairs (FL with VN and PC with GM); in cases of conflicting opinions, a third reviewer (AM) made the final decision. The protocol for this systematic review has been officially registered in the international PROSPERO (International Prospective Register of Systematic Reviews) database (CRD42025637441).

### Identification of Articles

The search was conducted in 3 main databases (PubMed, Scopus, and Web of Science) using structured queries. The full search strategy is available in Multimedia Appendix 2. The key terms included the following:

- “vaccin*,” “communication,” “literacy,” “hesitancy,” and “acceptance”
- References to tools and platforms such as “social media,” “chatbot,” and “television”

The use of Boolean operators (“AND,” “OR,” and “NOT”) allowed the combination of terms to maximize sensitivity and specificity.

The identified articles were downloaded and imported into the Rayyan software (Qatar Foundation) for screening. The search produced an initial total of 17,407 articles.

### Application of Restrictions

Several restrictions were progressively applied to refine the selection (Textbox 1).

**Applied restrictions.**

1. Temporal restriction (January 2014-October 2025)
  The selection focused on the last 10 years, including recent developments in technology and vaccination, especially after the COVID-19 pandemic.
2. Language restriction (English)
  Only articles published in English were included to ensure comparability and minimize interpretive ambiguity.
3. Exclusion of irrelevant types
  Reviews, letters, editorials, commentaries, and non–peer-reviewed articles were excluded.
4. Removal of duplicates.

### Application of Inclusion/Exclusion Criteria

The inclusion criteria were defined based on 4 main elements according to the PICO (Population, Intervention/Exposure, Comparison, and Outcome) framework:

- Population: Studies on adults (≥18 years) with a sample size larger than 1000.
- Intervention/exposure: Strategies designed to improve vaccine attitudes, increase vaccination adherence, and reduce hesitancy.
- Comparison: Studies comparing different communication strategies or using control groups.
- Outcome: Indicators such as vaccine attitudes, vaccination adherence, or vaccine hesitancy.

Only peer-reviewed studies were considered, including surveys, ecological, case-control, cross-sectional, cohort, prevalence, quasi-experimental, and randomized controlled trials (RCTs).

The exclusion criteria were defined as follows:

- Studies not conducted on humans or conducted exclusively in pediatric populations or among health care professionals.
- Reviews, abstracts, editorials, letters, preprints, and other article types not covered by the inclusion criteria.
- Articles not in English.
- Articles that do not concern vaccines or strategies aimed at improving adherence or communication in the vaccination context.

The review process was carried out based on titles and abstracts.

### Data Extraction

For the 99 articles, a rigorous full-text review process was subsequently conducted to confirm full compliance with the inclusion and exclusion criteria and, if confirmed, to proceed with the extraction of relevant information and compilation of a specifically prepared Microsoft Excel spreadsheet. The review and data extraction processes were performed by 2 reviewers (VN and FL) and subsequently checked by others (PC, GM, and AM). The information extracted included the following: title, authors, year of publication, country/context setting, methodology, objectives/purpose of the study, study population and sample size, vaccine, communication method and details, duration, main results, public health implications, and limitations.

### Risk of Bias Assessment

The risk of bias was assessed using tools appropriate to the design of individual studies. For cross-sectional studies, the Joanna Briggs Institute Critical Appraisal Checklist for analytical cross-sectional studies was used [27]. It consists of 8 key criteria evaluating aspects such as participant selection, measurement validity, confounding factors, and statistical analysis. For RCTs, risk of bias was evaluated using the RoB 2.0 (Risk-of-Bias tool for randomized trials), developed by Cochrane, to appraise 5 domains: randomization, deviations from intended interventions, missing outcome data, measurement of outcomes, and selection of reported results. Each domain was rated using signaling questions, leading to an overall judgment of “low risk,” “some concerns,” or “high risk” of bias [28].

In nonrandomized experimental studies, risk of bias was evaluated using the ROBINS-I (Risk of Bias in Nonrandomized Studies of Interventions) tool, which assesses 7 domains: confounding, selection of participants, classification of interventions, deviations from intended interventions, missing data, outcome measurement, and selection of reported results. Judgments were made for each domain to determine an overall risk-of-bias rating: low, moderate, serious, critical, or no information [29].

Quality appraisal was performed by 2 researchers (VN and FL), and any discrepancies were resolved by a third (AM). Three separate graphical representations of the risk-of-bias assessment were generated using the robvis tool [30].

### Quality Assessment

Eligible studies were subjected to a rigorous evaluation of methodological quality using the Modified Medical Education Research Study Quality Instrument (M-MERSQI) tool, a quantitative assessment comprising 12 items across 7 domains, with a total possible score of 100 [31]. Studies were categorized into 3 quality levels: low quality (scores ranging from 0 to 35), medium quality (scores ranging from 36 to 65), and high quality (scores ranging from 66 to 100).

### Data Analysis and Synthesis

Despite the presence of quantitative data in several included studies, a meta-analysis was deemed unfeasible due to substantial heterogeneity in study designs, populations, communication exposures, and outcome measures. Therefore, we conducted a narrative and tabular synthesis of the findings from the included studies, organized around key themes and study designs: the association between traditional and social media and vaccine adherence, and the role of sociodemographic factors. For each synthesis, studies were selected based on the type of communication intervention evaluated. Where available, regression coefficients, relative risks, or odds ratios with 95% CIs were tabulated and summarized through narrative synthesis. Outcomes not explicitly designated as primary or secondary for the purposes of this review were excluded from both the results table and the synthesis.

## Results

### Overview of the Included Studies

**Figure 1:**
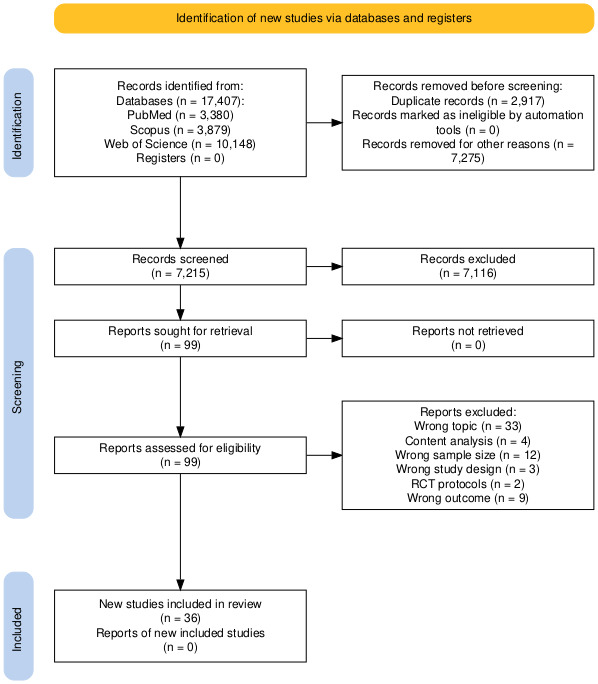
PRISMA (Preferred Reporting Items for Systematic Reviews and Meta-Analyses) flow diagram of the study selection process. The figure shows the number of records identified through database searching, along with the screening and eligibility stages, and the final number of studies included in the review. A total of 7275 records were excluded using database filters, and a further 7116 articles were excluded during title and abstract screening. RCT: randomized controlled trial.

At the end of the full-text review process (Figure 1), a total of 36 studies were included, all of which fully met the inclusion and exclusion criteria (see Tables 1-5) [32-67]. Among these, the vast majority were cross-sectional (n=26) [32-57], followed by nonrandomized controlled (quasi-experimental) studies (n=4) [60-63] and RCTs (n=4) [64-67]. One study was classified as a global cross-national analysis (n=1) [59], and another as a cohort study (n=1) [58]. Conversely, 63 studies were excluded for the following reasons: 33 either addressed vaccines without exploring constructs related to hesitancy, adherence, or attitudinal factors, or focused on communication channels and media not specifically evaluated within a vaccination context; 4 conducted content analyses of digital or social media platforms without considering a study population; 3 employed unsuitable study designs; 12 did not meet the sample size criterion; 2 were RCT protocols without reporting results; and 9 had irrelevant outcomes.

**Table 1:** Characteristics of cross-sectional studies on communication evidence and vaccination (n=26).

Study	Study design	Sample size	Country of study	Quality score (M-MERSQI^a^)	Vaccine discussed	Communication method	Main results
De Giorgio et al [32]	Cross-sectional study	1003 adult participants (median age 40 years) from Croatia, recruited via nonprobability snowball sampling.	Croatia	61	COVID-19	Multiple communication tools, including social network, television/radio, and internet forums	Participants informed via television/radio (odds ratio 2.35, 95% CI 1.71-3.23) or family doctors (odds ratio 2.53, 95% CI 1.78-3.61) were more likely to be vaccinated, whereas reliance on social networks (odds ratio 0.36, 95% CI 0.27-0.49) or blogs (odds ratio 0.34, 95% CI 0.22-0.52) reduced uptake.
Garg et al [33]	Cross-sectional study	Nationally representative sample of US adults from the Health Information National Trends Survey 6 survey; specific N varies per analysis (eg, millions weighted).	United States	67	Human papillomavirus	Health-related videos on social media	Increased exposure to health-related videos improved human papillomavirus vaccine awareness, with the strongest effects among adults aged 18-40 and higher education groups.
Garrett et al [34]	Cross-sectional study	1043 US adults, country-wide quota-based sample, ensuring data quality checks for online participation.	United States	61	COVID-19	Social media	TikTok users reported lower knowledge of COVID-19 guidelines, associated with greater vaccine hesitancy.
Hwang and Shah [35]	Cross-sectional study	4174 US parents of children under 18, including 138 parents with a childhood autism diagnosis, drawn from a nationwide survey.	United States	72	Vaccines in general	Various health information sources (eg, social media, magazines)	Valuing magazines was positively associated with perceived vaccination benefits; valuing television correlated with program maintenance; and social media use correlated negatively with perceived benefits.
Jia et al [36]	Cross-sectional study	1141 US adults; mean age 33.8 (SD 10.8; range 18-79) years; 55% male; 83% White.	United States	62	COVID-19	10 social media posts (from the CoVaxxy dataset, classified by Health Belief Model constructs)	Perceived barriers decreased vaccination intention (*b*=–0.12, *P*=.01), while cues to action (*b*=0.19, *P*=.002) and self-efficacy messages (*b*=0.06, *P*=.04) increased it. “Likes” mediated effects of susceptibility (*b*=0.03, *P*=.02) and cues to action (*b*=0.14, *P*=.01) on intentions. Celebrities generated more likes (*b*=0.04, *P*=.05) and politicians more shares (*b*=0.05, *P*=.03).
Jin et al [37]	Cross-sectional study	1946 internet users across Pakistan adults (54.83% men; 44.6% aged between 18 and 30) exposed to COVID-19 vaccine infodemics.	Pakistan	76	Polio	Public service advertisements and digital media platforms	Public service advertisements significantly increased polio vaccine acceptance among Pakistani parents, counteracting misinformation and religious fatalism.
Jung et al [38]	Cross-sectional study	Pooled data from 151,209 women (15+ with child <5 years) from 13 sub-Saharan African countries.	13 countries of Sub-Saharan Africa	75	Childhood vaccines in general	Traditional media (radio, television, and newspaper)	Among 151,209 women in sub-Saharan Africa, daily television/radio consumption was associated with higher childhood vaccination uptake (Bacillus Calmette-Guérin vaccine for tuberculosis; diphtheria, tetanus, and pertussis vaccine; polio; and measles).
Jung [39]	Cross-sectional study	1571 married women (aged between 20 and 40) with underage children from South Korea, China, and Japan (nationally representative online sample).	South Korea, China, and Japan	75	Bacillus Calmette-Guérin vaccine, diphtheria, tetanus, and pertussis vaccine, polio vaccine, and measles vaccine	Television, radio, book, and newspaper	Chinese and South Korean mothers using the internet were less likely to vaccinate children, while traditional media exposure increased uptake.
Khanijahani et al [40]	Cross-sectional study	Pooled data from 220,570 noninstitutionalized US adults over 7 years (2012-2018) from the National Health Interview Survey.	United States	72	Influenza	Internet use for health information and communication	US adults using the internet for health information were more likely to be vaccinated against influenza (odds ratio 1.52, 95% CI 1.45-1.59).
Kim and Jung [41]	Cross-sectional study	1367 Korean adults, nationally representative sample from a mixed method survey (face-to-face and web-based).	South Korea	72	Vaccines in general	General mass media (radio, newspaper, smartphone, and internet)	Higher socioeconomic background in South Korea correlated positively with vaccination adherence.
Kim et al [42]	Cross-sectional study	9584 community-dwelling Medicare beneficiaries (weighted N=50,029,030) from the Winter 2021 Medicare Current Beneficiary Survey.	United States	72	COVID-19	Informal sources of information (social media, internet, and friends/family) and formal source (traditional news, government guidance, and health care providers)	Reliance on informal sources reduced the likelihood of COVID-19 vaccination (odds ratio 0.65, 95% CI 0.56-0.75), testing (odds ratio 0.85, 95% CI 0.74-0.98), and preventive behaviors (odds ratio 0.61, 95% CI 0.50-0.74).
Kobayashi et al [43]	Cross-sectional study	10,192 users of the Corowa-kun chatbot in Japan (median age 55 years, 74% female), participating in an in-chatbot survey.	Japan	61	COVID-19	Corowa-kun chatbot, free chatbot in LINE	Use of the Corowa-kun chatbot revealed higher hesitancy among individuals aged 16-34 (odds ratio 3.7, 95% CI 3.0-4.6) and women (odds ratio 2.4, 95% CI 2.1-2.8).
Lv et al [44]	Cross-sectional study	1673 older adult participants (≥60 years) in Beijing, China, selected via multistage stratified random sampling.	China	72	Influenza	Television, community message boards, and doctors	Older adults exposed to television (odds ratio 1.40, 95% CI 1.12-1.75) and community bulletin boards (odds ratio 1.81, 95% CI 1.45-2.27) were more likely to receive influenza vaccination; coverage was higher in rural versus urban areas (odds ratio 2.57, 95% CI 1.80-3.66).
Melovic et al [45]	Cross-sectional study	1593 parents from Montenegro, Serbia, and Bosnia and Herzegovina responding to online questionnaires.	Montenegro, Serbia, and Bosnia and Herzegovina	68	Vaccines in general	Online media and social marketing	Online media in Montenegro, Serbia, and Bosnia-Herzegovina influenced parental attitudes and correlated with trust in vaccines.
Nah et al [46]	Cross-sectional study	1000 Cameroonians (43.8% female, mean age 20.74 years).	Cameroon	65	COVID-19	Social media use and medical mistrust	Social media use and medical mistrust were positively associated with misinformation beliefs and vaccine hesitancy.
Nah et al [47]	Cross-sectional study	1136 African Americans (53.5% female, age 19-87 years).	United States	65	COVID-19	Social media use and medical mistrust	Same trend observed among African Americans, with social media use and mistrust linked to misinformation and hesitancy.
Piltch-Loeb et al [48]	Cross-sectional study	2650 US individuals (18+) in vaccine priority groups, recruited via mobile survey platform (Pollfish RDE).	United States	72	COVID-19	Traditional media (television, newspapers, and radio) and social media (Facebook, Instagram, YouTube, Twitter, and TikTok)	High trust in television and newspapers increased COVID-19 vaccine acceptance, while reliance on social media or mixed sources decreased acceptance.
Recio-Román et al [49]	Cross-sectional study	27,524 individuals (15+ years) residing in European Union member states, from the EUROBAROMETRO 91.2 survey (2019).	European Union	79	Vaccines in general	Traditional media (television, radio, newspaper, magazine, social networks, internet sites, etc) and institution (political parties, regional or local public authorities, national government or parliament, European Union, etc)	In 27,524 Europeans, television (β=−.094), radio (β=–.066), and newspapers/magazines (β=−.194) reduced hesitancy; online media weaker (β=−.041); and lack of information increased hesitancy (β=.049).
Sakamoto et al [50]	Cross-sectional study	4062 Japanese university students, recruited via online questionnaire (Learning Management System).	Japan	58	COVID-19	Multiple communication tools. Specifically evaluated a 14-minute-long educational movie to provide information about the COVID-19 vaccine.	Japanese university students reported positive attitudes when informed via television/radio, while negative attitudes were more common among YouTube/Instagram users; the female gender was associated with positivity.
Sunohara et al [51]	Cross-sectional study	3348 controls (20-65 years) from Sapporo, Japan, matched for age and sex to the general population, without COVID-19 diagnosis.	Japan	72	COVID-19	Multiple communication tools (eg, television, newspapers, social media)	Lower odds of incomplete vaccination among television (odds ratio 0.31, 95% CI 0.21-0.44) and newspaper users (odds ratio 0.32, 95% CI 0.20-0.50); higher odds for Facebook (odds ratio 2.36, 95% CI 1.24-4.48) and blogs/bulletins (odds ratio 4.81, 95% CI 2.72-8.48). Older age and male gender were linked to lower acceptance.
Teoh et al [52]	Cross-sectional study	1037 young adults (18-26 years) at the Minnesota State Fair, convenience sample (predominantly White, educated, insured, and vaccinated).	United States	61	Human papillomavirus	Graphic messages: 8 Instagram graphic mockups promoting human papillomavirus vaccination to prevent cancer (4 categories: infographics, disease photos, young adult cancer patient photos, and humorous graphics).	The Instagram human papillomavirus campaign showed modest demographic differences; women rated patient-photo graphics more favorably.
Thompson et al [53]	Cross-sectional study	1192 parents of children/adolescents (9-17 years) from 13 North Texas counties, via web-based surveys.	United States	61	Human papillomavirus	Social media, trust in providers, internet verification skills, and demographics	Fathers had a lower likelihood of human papillomavirus vaccine acceptance than mothers (adjusted odds ratio 0.42, 95% CI 0.27-0.64).
Wada and Smith [54]	Cross-sectional study	3140 Japanese individuals (20-69 years), recruited from an online survey company.	Japan	61	Vaccines in general	Health care providers, national or local government, family, friends, television, newspapers, internet, and books	Women trusted family (odds ratio 1.60), newspapers (odds ratio 1.56), internet (odds ratio 2.19), and books (odds ratio 2.99) more; men trusted friends (odds ratio 1.96).
Wamba et al [55]	Cross-sectional study	3108 adults from France (2001) and South Africa (1107), members of a market research firm’s panel.	France and South Africa	75	COVID-19	Traditional media, specialized health sources, family or friends, social media, and web-based sources	Identified 4 opinion profiles; enthusiasts, sceptics, and followers trusted traditional and health sources; conspiracy theorists showed low trust in all channels. Social media influence did not differ by profile.
Yoneoka et al [56]	Cross-sectional study	30,053 participants in a nationwide online survey in Japan (February to March 2021), using quota sampling (matched for age, gender, prefecture population).	Japan	61	COVID-19	Multiple media channels (ie, medical professionals, television, newspapers, The Novel Coronavirus Expert Meeting, and local government)	Uncertain individuals avoided authoritative sources; reliance on expert meetings/local government associated with unwillingness, possibly due to perceived pressure. Under 50s sought authoritative information; over 50s relied more on social media.
Al Zahrani [57]	Cross-sectional study	7159 adults (18+) living in Saudi Arabia.	Saudi Arabia	61	Vaccines in general	Promotion by health care leaders	Men were more responsive than women to health care leaders’ influence in promoting vaccination.

^a^M-MERSQI: Modified Medical Education Research Study Quality Instrument.

**Table 2:** Characteristics of cohort studies on communication evidence and vaccination (n=1).

Study	Study design	Sample size	Country of study	Quality score (M-MERSQI^a^)	Vaccine discussed	Communication method	Main results
DeMora et al [58]	Cohort study	1768 Americans from a probability-based longitudinal survey conducted over 10 months.	United States	71	COVID-19 and influenza	Social media	Social media engagement increased vaccination rates, with effects moderated by political orientation (Democrats: pathogen information exposure; Republicans: perceived peer vaccination).

^a^M-MERSQI: Modified Medical Education Research Study Quality Instrument.

**Table 3:** Characteristics of global cross-national analysis on communication evidence and vaccination (n=1).

Study	Study design	Sample size	Country of study	Quality score (M-MERSQI^a^)	Vaccine discussed	Communication method	Main results
Wilson and Wiysonge [59]	Global cross-national analysis	Examination of public attitudes on vaccine safety in 137 countries, vaccination rates in 166 countries, and 258,769 geocoded vaccination-related tweets	Worldwide	64	Vaccines in general	Social media	Significant relationship between the use of social media for organizing offline action and public doubts about vaccine safety. Substantial connection between foreign disinformation campaigns and declining mean vaccination rates over time.

^a^M-MERSQI: Modified Medical Education Research Study Quality Instrument.

**Table 4:** Characteristics of nonrandomized controlled studies (quasi-experimental) on communication evidence and vaccination (n=4).

Study	Study design	Sample size	Country of study	Quality score (M-MERSQI^a^)	Vaccine discussed	Communication method	Main results
Evans et al [60]	Nonrandomized controlled study (quasi-experimental)	1933 Nigerians (adults 18+) from all 37 states, recruited via Facebook, followed for 10 months.	Nigeria	69	COVID-19	Social media (Facebook/Instagram)	Facebook/Instagram campaign in Nigeria increased COVID-19 vaccination rates compared with control participants.
Kim et al [61]	Nonrandomized controlled study (quasi-experimental)	5804 respondents from Tanzania (3442 before campaign and 2362 after campaign), convenience sample.	Tanzania	58	COVID-19	“The One by One: Target COVID-19” social media campaign. High-profile and high-impact influencers and viral content on social media platforms	The Tanzanian social media campaign reduced hesitancy in adults ≥35 years and increased uptake in those aged 25-34, but showed no effect among 18-24-year-olds.
Pavoncello et al [62]	Cross-sectional study	854 adults at phase 1 survey (48.1% male and 51.9% female), 1034 adults at phase 2 survey; total vaccine doses delivered were 24,888.	Madagascar	58	COVID-19	“CoBoGo” campaign (multiple communication tools) using tailored adaptive strategy	Weekly vaccine uptake rose by 8% in phase 2 (relative risk 1.08; 95% CI 1.01-1.15). Vaccine hesitancy was unchanged (Δ=0.02; 95% CI –0.04 to 0.08), knowledge of reliable sources improved (Δ=0.04; 95% CI 0.003-0.08), perception of insufficient information decreased (Δ=–0.11; 95% CI –0.15 to –0.07), and recognition of community health workers increased (Δ=0.07; 95% CI 0.02-0.12).
Schuh et al [63]	Nonrandomized controlled study (quasi-experimental)	13,889 participants in a nationally representative quasi-experimental study in the United States, recruited via RIWI RDIT^b^ technology.	United States	63	COVID-19	Informational animated videos, with online experiments testing debunking/prebunking texts addressing messenger RNA vaccine misinformation versus control texts.	COVID-19 educational videos with ethnically congruent protagonists increased the likelihood of full viewing (odds ratio 1.89; *P*<.01).

^a^M-MERSQI: Modified Medical Education Research Study Quality Instrument.

^b^RIWI RDIT: Real-Time Interactive World-Wide Intelligence Random Domain Intercept Technology.

**Table 5:** Characteristics of RCTs^a^ on communication evidence and vaccination (n=4).

Study	Study design	Sample size	Country of study	Quality score (M-MERSQI^b^)	Vaccine discussed	Communication method	Main results
Daley et al [64]	Three-arm RCT	1093 pregnant women (≥18 years) with internet access and health insurance, followed until their child was 12-15 months.	United States	67	Vaccines in general	Website with vaccine information and social media components (vaccine social media arm); website with vaccine information only (vaccine information arm)	Both website-based strategies (with/without social media components) significantly improved parental vaccination attitudes and reduced concerns compared with usual care.
Geniole et al [65]	RCT	1584 UK residents (67.6% women; mean age 34.91 years) recruited via Prolific (online labor market).	United Kingdom	61	COVID-19	Internet memes	Exposure to pro-vaccination memes modestly but significantly increased COVID-19 vaccination intention by approximately 3.3 points on a 100-point scale, independent of age, gender, and political orientation. The effect was context-dependent—robust before the announcement of a safe/effective vaccine (~7-point increase) but negligible thereafter—with no corresponding pre/postannouncement change in the control group.
Lee et al [66]	RCT	2045 adult guardians of unvaccinated children and seniors in Thailand, Hong Kong, and Singapore, with 748 participants completing the study after follow-up.	Thailand, Hong Kong, and Singapore	63	COVID-19	COVID-19 chatbots (ChatSure or D24H via Messenger/WhatsApp)	COVID-19 chatbot increased acceptance and trust, particularly among minorities and lower-education groups.
Schmid et al [67]	RCT	Experiment 1: 1382 German adults; experiment 2: 1054 unvaccinated German adults, both recruited via the online panel provider respondi.de using quota sampling.	Germany	60	COVID-19	Online experiments testing debunking/prebunking texts addressing messenger RNA vaccine misinformation versus control texts	Religiosity moderated credibility judgments of debunking messages at the 2-month follow-up, whereas prebunking was not affected by spirituality.

^a^RCT: randomized controlled trial.

^b^M-MERSQI: Modified Medical Education Research Study Quality Instrument.

The publication period spanned from 2015 to 2024. Notably, the highest number of studies were published in 2022 (n=8) [42,43,50,53,56,60,63,66] and 2023 (n=8) [32,37,40,45,48,52,59,67], followed by 2024 (n=6) [35,38,39,41,44,54], 2018 (n=3) [33,34,61], 2021 (n=2) [46,49], 2020 (n=2) [51,55], 2025 (n=2) [64,65], and 2015 (n=2) [36,62]. A single study was published in each of the following years: 2019 [58], 2017 [47], and 2016 [57]. The studies originated from several countries: the United States accounted for the largest number (n=13) [33-36,40,42,47,48,52,53,58,63,64], followed by multicountry studies (n=7) [43,45,50,51,54,56,66] and Japan (n=5) [38,39,49,55,59]. Croatia [32], China [44], Cameroon [46], Germany [67], Nigeria [60], Pakistan [37], United Kingdom [65], South Korea [41], Saudi Arabia [57], Madagascar [62], and Tanzania [61] each contributed 1 study.

The included studies evaluated different vaccines. The COVID-19 vaccine was the most studied (n=19) [32,35,37-41,43-45,48,50,52-54,56,59,66,67], followed by studies on multiple vaccines (n=11) [33,34,36,47,57,58,60-62,64,65]. Vaccines for human papillomavirus (n=3) [42,46,63], influenza (n=2) [49,51], and polio (n=1) [55] were less represented.

Regarding sample size, 4 studies involved more than 10,000 participants [43,49,56,63], while 3 included more than 100,000 participants [38,40,59]. All remaining studies included more than 1000 participants [32-37,39,41,42,44-48, 50-55,57,58,60-62,64-67].

The overall methodological quality was assessed as “moderate” for 19 studies [32,34,36,43,46,47,50,52-54,56,57,59, 61-63,65-67] and “high” for the remaining 17 [33,35,37-42,44,45,48,49,51,55,58,60,64]. The M-MERSQI score for each study is provided in Tables 1-5.

**Figure 2:**
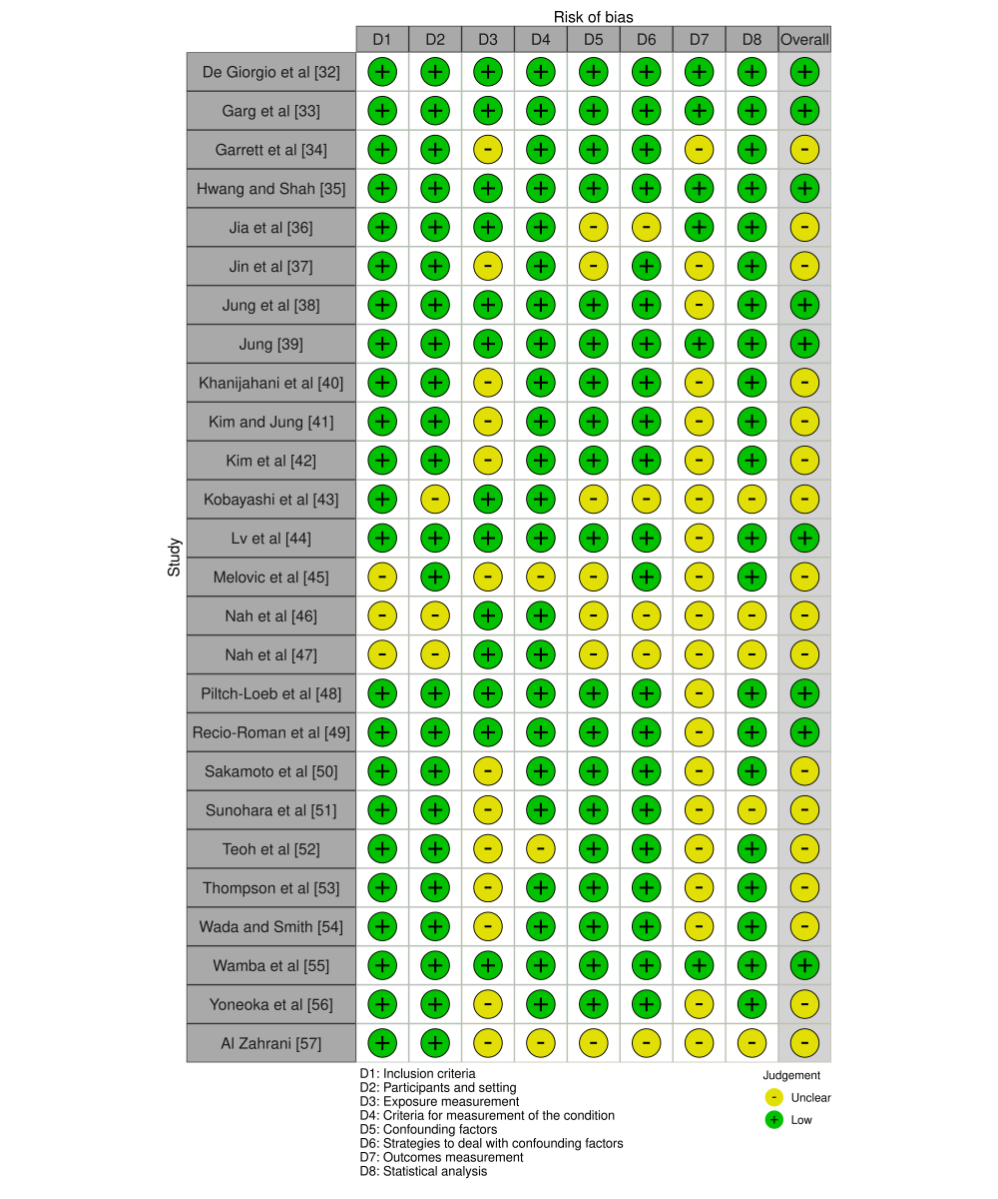
Risk of bias across cross-sectional studies (traffic-light plot). Domains assessed were as follows: D1, inclusion criteria; D2, description of participants and setting; D3, exposure measurement; D4, criteria for measuring the condition; D5, identification of confounders; D6, strategies to address confounding; D7, outcome measurement; and D8, statistical analysis. Each row represents an included study (n=26), and the rightmost column shows the overall study-level judgment derived from the domain ratings.

**Figure 3:**
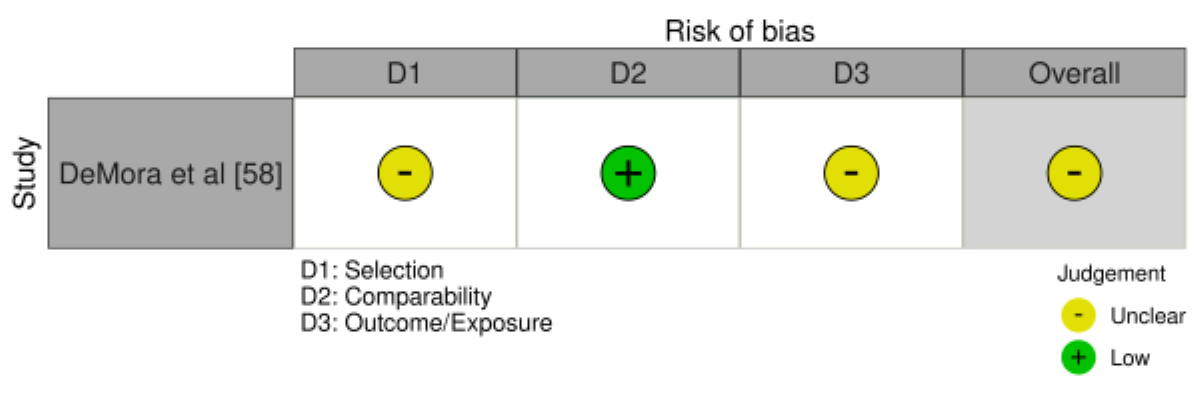
Risk of bias assessment for cohort studies (traffic-light plot). Domains assessed were as follows: D1, selection; D2, comparability; and D3, outcome/exposure. The overall risk-of-bias judgment is shown in the rightmost column.

**Figure 4:**
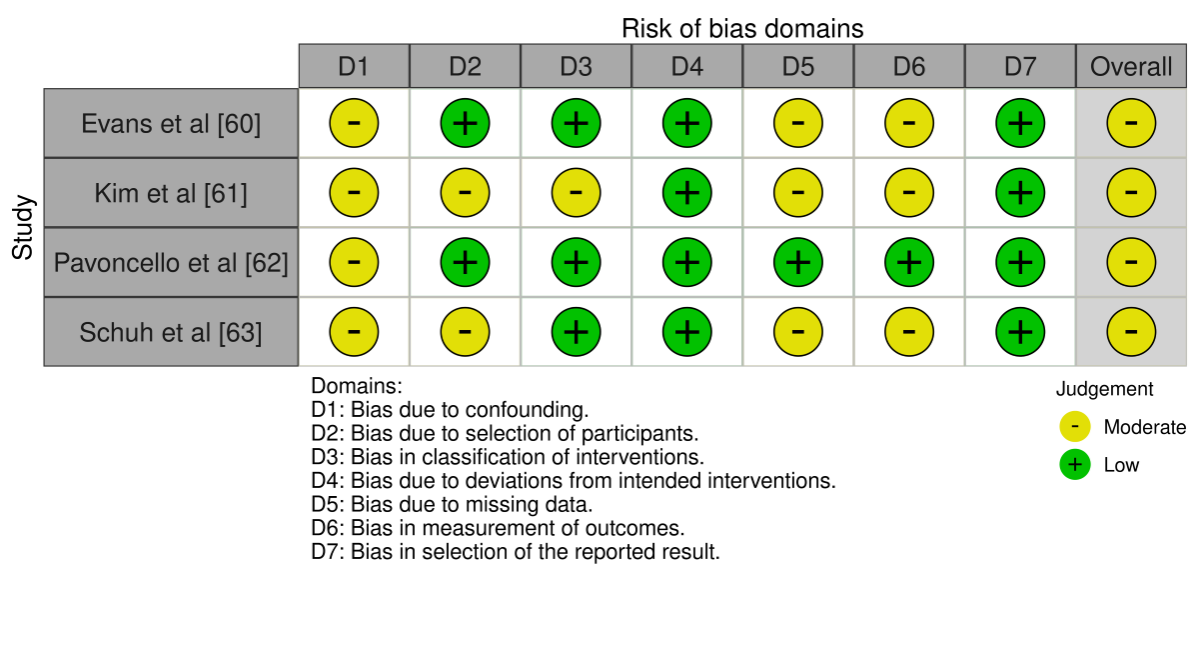
Risk of bias across included nonrandomized studies (traffic-light plot). Domains assessed were as follows: D1, bias due to confounding; D2, bias due to selection of participants; D3, bias in classification of interventions; D4, bias due to deviations from intended interventions; D5, bias due to missing data; D6, bias in measurement of outcomes; and D7, bias in selection of the reported result. The overall risk-of-bias judgment is shown in the rightmost column.

Across the cross-sectional studies, the risk of bias was generally low for selection/context (D1-D2), case definition (D4), control of confounders (D5-D6), and analytic appropriateness (D8); the most frequent limitations were exposure and outcome measurement (D3 and D7), due to self-report and limited external validation. Overall, 9 studies had a low risk of bias [32,33,35,38,39,44,48,49,55], and 17 had a moderate risk of bias [34,36,37,40-43,45-47,50-54,56,57] (Figure 2; also see [32-57]). The only cohort study achieved good comparability but had an unclear risk for selection and outcome assessment (Figure 3; also see [58]). The nonrandomized experimental studies were judged to have an overall moderate risk of bias. This determination was driven primarily by bias due to confounding (D1), rated as moderate across all studies, and by missing data (D5) and incomplete outcome data (D6), which were rated as moderate in 3 of the 4 studies [60,61,63]. Overall, no study was judged to have a serious risk of bias (Figure 4; see also [60-63]).

**Figure 5:**
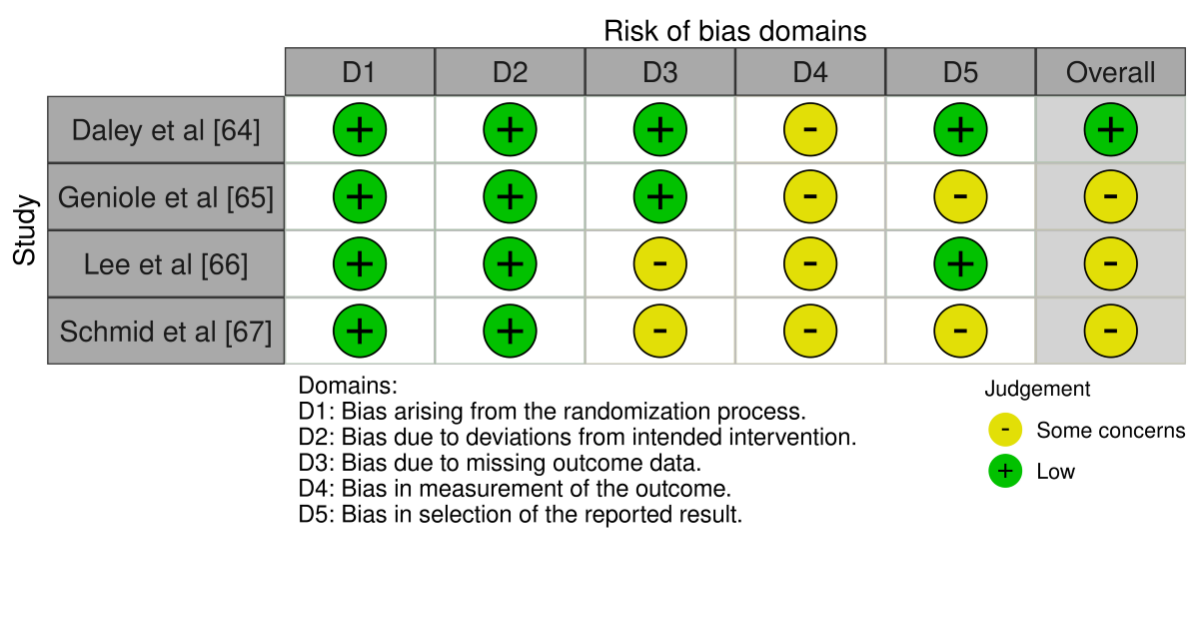
Risk of bias in randomized controlled trials (traffic-light plot). Domains assessed were as follows: D1, bias arising from the randomization process; D2, bias due to deviations from intended interventions; D3, bias due to missing outcome data; D4, bias in measurement of the outcome; and D5, bias in selection of the reported result. Each row represents an included RCT (n=4), with the overall risk-of-bias judgment shown in the rightmost column.

Finally, RCTs showed a low risk of bias for randomization and intervention fidelity, with the main concerns related to outcome measurement and missing data. Selective reporting was occasionally unclear. Overall, most trials had some concerns (Figure 5; see also [64-67]).

### Observational Evidence About Media Exposure and Vaccine Attitudes, Acceptance, and Hesitancy

Sixteen cross-sectional studies [32,34-40,42,44,45,48-51,56] and 1 global cross-national analysis [59] examined the effect of media on vaccination acceptance. Recio-Román et al [49], in a study of 27,524 European citizens, underscored a pronounced positive correlation between traditional media and the mitigation of vaccine hesitancy, compared with new media. Television (β=−.094; *P*<.001; 95% CI −0.132 to −0.056), radio (β=−.066; *P*<.001; 95% CI −0.092 to −0.041), and newspapers/magazines (β=−.194; *P*<.001; 95% CI −0.229 to −0.162) exerted considerable influence. The effect on vaccine adherence was mainly mediated by reductions in hesitancy, except for television, which did not show a significant effect on increasing vaccine uptake, potentially due to the context and sociodemographic characteristics of the recipients. Conversely, online media demonstrated less influence in reducing hesitancy (β=−.041; *P*<.001; 95% CI −0.068 to −0.014) and indirect influence in increasing adherence (β=.021; *P*<.05; 95% CI 0.003-0.038), while other internet sources showed no statistical significance. Lack of information increased vaccine hesitancy (β=.049; *P*<.001; 95% CI 0.008-0.089) and decreased vaccine adherence (β=−.063; *P*<.001; 95% CI −0.090 to 0.037), highlighting the detrimental effects of unreliable communication and information. Similar findings were reported by Sakamoto et al [50], who observed positive attitudes toward COVID-19 vaccination among 4062 Japanese university students, particularly among those relying on television and radio, whereas negative attitudes were more frequent among users of YouTube and Instagram. Likewise, Jung et al [38], in a study involving 151,209 women from sub-Saharan African countries, showed that daily exposure to television and radio increased the likelihood of childhood vaccination against tuberculosis (Bacillus Calmette-Guérin); diphtheria, tetanus, and pertussis; polio; and measles. Similarly, Jung [39] demonstrated that radio use was significantly associated with a higher likelihood of vaccinating children (adjusted odds ratio 1.577, 95% CI 1.040-2.393; *P*<.05) among Japanese, Chinese, and South Korean mothers. Conversely, internet use in China and South Korea was associated with a lower likelihood of vaccinating children.

Hwang and Shah [35] found that the value attributed to magazines by 4174 US parents was positively associated with their perception of vaccination benefits. Additionally, the value attributed to television was positively correlated with the maintenance of vaccination programs. By contrast, social media use was negatively associated with the perception of vaccination benefits. De Giorgio et al [32] highlighted that participants informed by television, radio (odds ratio 2.35, 95% CI 1.71-3.23; *P*<.001), or family doctors (odds ratio 2.53, 95% CI 1.78-3.61; *P*<.001) were more likely to be vaccinated, whereas reliance on social networks (odds ratio 0.36, 95% CI 0.27-0.49; *P*<.001), general internet forums/blogs (odds ratio 0.34, 95% CI 0.22-0.52; *P*<.001), or friends/acquaintances (odds ratio 0.66, 95% CI 0.48-0.91; *P*=.01) was linked to lower uptake. Lv et al [44] found that older adults receiving information through television (odds ratio 1.403, 95% CI 1.124-1.752; *P*<.01) and community bulletin boards (odds ratio 1.812, 95% CI 1.446-2.270; *P*<.01) were more likely to be vaccinated against influenza. Similarly, Kim et al [42] showed that adults using informal sources were less likely to receive the COVID-19 vaccine (odds ratio 0.65, 95% CI 0.56-0.75), undergo COVID-19 testing (odds ratio 0.85, 95% CI 0.74-0.98), or adopt preventive behaviors (odds ratio 0.61, 95% CI 0.50-0.74), whereas reliance on diversified sources increased all 3 outcomes.

The negative role of social media was further highlighted by Garrett et al [34], who found lower knowledge of COVID-19 guidelines among TikTok users (β=–.29; 95% CI –0.58 to –0.004; *P*=.047). Instagram (β=–.40; 95% CI –0.68 to –0.12; *P*=.005) and Twitter (β=–.33; 95% CI –0.58 to –0.08; *P*=.01) use was significantly associated with perceiving the guidelines as overly strict. Additionally, Facebook (β=–.23; 95% CI –0.42 to –0.043; *P*=.02) and TikTok (β=–.25; 95% CI –0.50 to –0.009; *P*=.04) use was linked to lower adherence to the guidelines. Among Cameroonians and African Americans, social media use, along with medical mistrust, was positively associated with beliefs in misinformation about the COVID-19 vaccine and, inferentially, with vaccine hesitancy [46,47]. Wilson and Wiysonge [59], in a global cross-national observational study using survey data from 137 countries (Wellcome Global Monitor), vaccination rates from 166 countries (WHO), and 258,769 geocoded tweets (2018-2019), showed that social media use for organizing action was significantly associated with doubts about vaccine safety. Foreign disinformation campaigns were linked to declining vaccination rates (≈2% yearly decrease per scale point) and a 15% increase in negative vaccine-related tweets.

Piltch-Loeb et al [48] illustrated that local television was the most commonly used source of COVID-19 vaccine information (61.0%). National television (relative risk reduction 1.25; 95% CI 1.02-1.53; *P*<.05), local television (relative risk reduction 1.75; 95% CI 1.40-2.19; *P*<.001), and national newspapers (relative risk reduction 1.81; 95% CI 1.45-2.24; *P*<.001) were associated with higher acceptance, particularly when supported by high levels of trust. Acceptance significantly decreased among individuals relying on social media (relative risk reduction 0.45; 95% CI 0.32-0.64; *P*<.001) or on both social and traditional media (relative risk reduction 0.81; 95% CI 0.66-1.00; *P*=.05). Sunohara et al [51] reported lower odds of incomplete second vaccinations among television (odds ratio 0.31, 95% CI 0.21-0.44) and newspaper users (odds ratio 0.32, 95% CI 0.20-0.50), and higher odds among those relying on books (odds ratio 3.34, 95% CI 1.58-7.06), commercial video sites (odds ratio 2.22, 95% CI 1.44-3.43), Facebook (odds ratio 2.36, 95% CI 1.24-4.48), and personal blogs or electronic bulletin boards (odds ratio 4.81, 95% CI 2.72-8.48).

Likewise, Yoneoka et al [56] noted that 30,053 Japanese individuals who were hesitant about vaccination often avoided authoritative sources such as medical professionals, the government, and television. Jin et al [37] showed that public service advertisements positively influenced offspring polio vaccine acceptance among 2160 Pakistani parents, counteracting misinformation, fake news, and religious fatalism.

However, the positive and responsible use of the internet and social media can be a valuable strategy for disseminating high-quality information and increasing trust in vaccines, as highlighted by Khanijahani et al [40]. Adults using the internet for health-related information were more likely to receive influenza vaccinations (odds ratio 1.52, 95% CI 1.45-1.59) compared with non–health information seekers and noninternet users. Melovic et al [45], evaluating 1593 participants in Montenegro, Serbia, and Bosnia-Herzegovina, demonstrated that online media play a substantial role in shaping parents’ attitudes toward child vaccination and correlate with the degree of trust in vaccines. Jia et al [36] tested how Health Belief Model constructs and social media messaging affect COVID-19 vaccine intentions among 1141 unvaccinated US adults. Perceived barriers were negatively (*b*=–0.12; *P*=.01) and cues to action positively (*b*=0.19; *P*=.002) associated with vaccination intention; self-efficacy messages also increased intention (*b*=0.06; *P*=.04). Engagement, particularly “likes,” significantly mediated the effect of susceptibility and cues to action on both vaccination intention and persuading others.

### Observational Evidence About the Role of Sociodemographic Factors

A total of 15 cross-sectional studies [32,33,37,41,43,44,49-57] and 1 cohort study [58] addressed the role of sociodemographic factors. Recio-Román et al [49] showed that older age groups had lower odds of vaccination than younger ones, while no statistically significant differences were observed after adjusting for occupation. Higher educational attainment, better economic circumstances, and higher social class were associated with greater uptake. Families with 1 child were more likely to vaccinate, whereas no statistically significant differences were found for families with more than 1 child. Political orientation also mattered, with left-leaning individuals showing 8.7% higher odds of vaccination compared with centrists. Garg et al [33] demonstrated greater awareness of the human papillomavirus vaccine among US adults exposed to health-related videos on social media, particularly among younger and more educated adults (18-40 years). De Giorgio et al [32] confirmed that individuals with a bachelor’s degree (odds ratio 2.25, 95% CI 1.14-4.46; *P*=.02) or a PhD (odds ratio 1.97, 95% CI 1.01-3.52; *P*=.02) were more likely to receive COVID-19 vaccination compared with those with only a high school education.

Age differences also played a role. Yoneoka et al [56] reported that uncertain individuals aged under 50 years typically sought vaccine information from authoritative sources, including health care professionals, expert meetings, and both local and central government agencies. By contrast, individuals aged over 50 years relied more on social media. However, Kobayashi et al [43] found that young adults (odds ratio 3.7, 95% CI 3.0-4.6), aged 16-34 years, and women (odds ratio 2.4, 95% CI 2.1-2.8) were more likely to develop hesitancy when exposed to chatbot-based COVID-19 vaccine information. Sunohara et al [51] also documented lower acceptance among older individuals and males using social media.

Regarding gender, Teoh et al [52] examined the appeal of different graphic formats for human papillomavirus vaccine promotion among 1037 young adults (aged 18-26 years) in Minnesota. They found significant differences, albeit modest in effect size, related to demographic characteristics such as gender, ethnicity, and level of digital health literacy. Women rated patient photos higher than men (mean difference 0.28; 95% CI 0.14-0.41; *P*=.0001). Participants with high eHealth literacy provided higher ratings for infographics, humorous graphics, and disease photos. Sakamoto et al [50] also highlighted that female gender was associated with a positive attitude toward the vaccine. Wada and Smith [54] illustrated gender differences related to trust in government recommendations and perceived reliability of various information sources. Specifically, women tended to consider information from family (odds ratio 1.60, 95% CI 1.23-1.99), newspapers (odds ratio 1.56, 95% CI 1.03-2.15), the internet (odds ratio 2.19, 95% CI 1.58-2.73), and books (odds ratio 2.99, 95% CI 2.19-3.40) more reliable, whereas men tended to place more trust in information from friends (odds ratio 1.96, 95% CI 1.24-2.60). Conversely, Al Zahrani [57] highlighted that men were significantly more inclined than women to accept the influence of health care leaders in promoting vaccination. Thompson et al [53] found that fathers had a significantly lower likelihood of accepting human papillomavirus vaccination compared with mothers (adjusted odds ratio 0.42, 95% CI 0.27-0.64; *P*<.001). Regarding contextual differences, Lv et al [44] found that vaccination coverage among the older adult population in Beijing was significantly higher in rural areas (odds ratio 2.57, 95% CI 1.801-3.655; *P*<.01) compared with urban areas.

DeMora et al [58] demonstrated that the relationship between social media use and vaccine uptake was shaped by political orientation, being linked to information about emerging pathogens among Democrats and to the belief that individuals within their social circle are receiving the recommended vaccines among Republicans.

Beliefs and opinions play a critical role in influencing vaccine acceptance. Wamba et al [55] identified 4 opinion profiles: enthusiasts, skeptics, followers, and conspiracy theorists. Enthusiasts, followers, and skeptics generally placed greater trust in traditional media, friends and family, online resources, and specialized health sources, whereas conspiracy theorists demonstrated low trust in all these sources. Social media use showed no significant differences across sociodemographic profiles, suggesting that it is not the primary channel of influence. Furthermore, Jin et al [37] suggested that religious fatalism significantly moderated the association between risk perception and polio vaccine acceptance, showing an inverse effect (β=−.27; *P*=.01). Kim and Jung [41] highlighted that higher socioeconomic status facilitated access to reliable information channels, supporting vaccination adherence.

### Experimental Evidence About Media Exposure and Vaccine Attitudes, Acceptance, and Hesitancy

A randomized controlled trial [64] and 2 nonrandomized experimental studies [60,62] provided particularly strong evidence on the relationship between media exposure and vaccine acceptance.

Pavoncello et al [62], in their quasi-experimental study, evaluated the CoBoGo campaign in Madagascar (based on radio spots, community-based initiatives, and endorsement by community health workers), which applied a tailored, adaptive strategy. Phase 2 adaptations produced a significant 8% weekly increase in uptake (relative risk 1.08; 95% CI 1.01-1.15). Vaccine hesitancy remained stable (Δ=0.02; 95% CI –0.04 to 0.08), while sustainability improved through the training of 340 staff, facility upgrades, and integration of standard operating procedures. In an RCT by Daley et al [64], 1093 US pregnant women were randomized to one of the following: a website with vaccine information and social media components; a website with vaccine information only; or usual care. When comparing baseline with time point 1 among vaccine-hesitant parents, both strategies were associated with significant improvements in vaccination attitudes relative to the usual care group. Similarly, when comparing baseline with time point 2, both arms were linked to significant reductions in parental concerns about vaccination.

Similarly, Evans et al [60] showed that Nigerian adults exposed to a public health campaign on Facebook and Instagram exhibited higher vaccination rates. At the first follow-up, the vaccination rate was 31.7% in the treatment states compared with 25.3% in the comparison states, with an adjusted difference of 7.8 percentage points (*P*=.02). By the second follow-up, vaccination rates had increased to 44.1% in the treatment states and 35.0% in the comparison states, with a nonsignificant adjusted difference of 11.0 percentage points (*P*=.07).

### Experimental Evidence About the Role of Sociodemographic Factors

Three RCTs [65-67] provided significant evidence on the role of sociodemographic determinants. Schmid et al [67], in a large-scale RCT, demonstrated that at 2-month follow-up, religiosity exerted a strong moderating effect on individuals’ credibility judgments of a false myth–debunking message regarding messenger RNA vaccines (*b*=0.17; 95% CI 0.05-0.30; t_810_=2.80; *P*=.005), whereas no such effect was observed in the short term (*b*=0.01; 95% CI −0.09-0.10; t_1377_=0.10; *P*=.92). By contrast, the effectiveness of a prebunking message appeared to be unaffected by spirituality (*b*=0.04; 95% CI –0.05 to 0.14; t_1049_=0.89; *P*=.37). Beyond religiosity, another RCT emphasized the role of education. Lee et al [66], testing the effectiveness of a COVID-19 chatbot among unvaccinated or hesitant individuals, observed the greatest improvements in vaccine acceptance and trust among participants belonging to minority groups and those with lower levels of education. Geniole et al [65] showed that exposure to provaccination memes modestly but significantly increased COVID-19 vaccination intention by about 3.3 points on a 100-point scale, independent of age, gender, and political orientation. The effect was context-dependent—robust before the announcement of a safe and effective vaccine (~7-point increase) but negligible thereafter—with no corresponding pre/postannouncement change in the control group. Regarding age, Kim et al [61] highlighted that a social media campaign aimed at boosting COVID-19 vaccination confidence in Tanzania increased uptake mainly among individuals aged 25-34 years, with limited effects in younger groups. A significant reduction in vaccine hesitancy was observed among individuals aged 35 years and older. Regarding the role of cultural adaptation, Schuh et al [63] showed that ethnic congruence between the protagonist of educational videos about the COVID-19 vaccine and the viewer increased the likelihood of watching the video in full (odds ratio 1.89; *P*<.01).

## Discussion

### Overview and Rationale

Amid the COVID-19 “infodemic,” understanding how information delivery is associated with vaccine literacy and public trust has become a critical public health priority. Therefore, this systematic review analyzes the correlation between communication media and vaccination attitudes, intentions, and hesitancy across different study designs, while specifically examining the moderating influence of sociodemographic determinants.

### Main Findings

The review synthesizes evidence from 36 studies, predominantly cross-sectional, showing that tailored communication strategies across both digital and traditional media channels play a significant role in enhancing vaccine acceptance and mitigating hesitancy. While adaptive digital interventions and social media campaigns are particularly effective in reaching specific cohorts, such as hesitant populations and individuals from minority or lower-education backgrounds, traditional media appear to be associated with higher levels of public trust and adherence. Furthermore, the efficacy of these public health messages is strongly moderated by sociodemographic and cultural factors, including religiosity and cultural congruence, which influence message credibility and engagement.

### Cross-Sectional Evidence: Patterns and Sociodemographic Moderation

The synthesis of cross-sectional evidence suggests that exposure to traditional media is strongly associated with reduced vaccine hesitancy and higher uptake rates across large-scale studies, likely due to the editorial oversight inherent in these channels [68-70]. Conversely, social media use is frequently observed as a major vehicle for antivaccination narratives and conspiracy theories, exacerbated by the echo chamber effect [71-73]. However, this relationship appears ambivalent [74]: purposeful internet use for health-seeking behavior remains a positive predictor of vaccination [40], suggesting that user intent moderates the medium’s effect.

Across sociodemographic lines, high educational attainment and socioeconomic status generally appear to buffer against hesitancy. Complex influences of age and gender are observed. Young adults appear to prefer official and verified information sources, whereas older adults rely more heavily on social media, reflecting the “digital divide” phenomenon [75]. Furthermore, older individuals may have concerns regarding potential interactions between vaccines and preexisting conditions [76]. Regarding gender, women were found to prefer official sources of vaccine information, suggesting a greater reliance on messages emphasizing family or community protection [77]. Nevertheless, previous studies indicate that women are also more likely to delay or refuse vaccination due to fear of side effects and increased exposure to antivaccine content focusing on maternal health and fertility [78,79]. Cultural factors were observed to significantly shape trust in information sources, indicating that messaging must be contextually adapted [70,80]. The validity of these findings is supported by large sample sizes and generally moderate-to-high quality scores, but the cross-sectional design precludes causal inference, limiting conclusions to associations rather than direct effects. It is important to note that 17 out of 26 studies [34,36,37,40-43,45-47,50-54,56,57] have a moderate risk of bias, primarily due to measurement error, as most studies rely on self-reported exposure and vaccination status, potentially introducing recall and social desirability biases.

### Longitudinal Cohort Study and Global Analysis

The review goes beyond cross-sectional data by including 1 cohort study [58] and 1 global cross-national analysis [59]. The global study of 137 countries showed that foreign disinformation campaigns are strongly associated with declining vaccination rates, with an estimated 2% annual decrease in coverage for each increase in disinformation, alongside a 15% increase in negative vaccine-related tweets.

The cohort study, based on a 10-month US survey, showed that social media engagement could increase vaccination uptake, but only when moderated by political orientation. Democrats respond more to information about emerging health threats, whereas Republicans are influenced by perceptions of peer vaccination [58,59]. These results are adequately controlled for the main confounding factors (eg, sociodemographic factors), but a residual risk of bias remains due to self-reported outcomes and potential inconsistencies with selection criteria. Together, these studies suggest that social media can promote vaccination through normative influence but remains highly vulnerable to disinformation and external manipulation.

### Experimental Evidence: Validating Targeted Interventions

While observational studies suggest a negative correlation between general social media use and vaccine trust, experimental findings confirm that well-structured communication campaigns (based on traditional media such as radio, community-based initiatives, and the active involvement of trusted health care professionals, local institutions, and well-known testimonials) can effectively reverse this trend, depending on design, composition, and content quality [60,62,64]. These results are supported by low outcome measurement bias, as they are based on objective administrative data. This aligns with the example of the Mediterranean diet, which illustrates how well-coordinated communication campaigns can significantly shape health-related behaviors [81,82]. Furthermore, digital tools can bridge equity gaps noted in cross-sectional sociodemographic analyses, as shown by Lee et al [66] regarding minorities and individuals with lower socioeconomic status and education levels, who are more vulnerable to inequities and distrust toward scientific sources [83,84]. Similarly, creative communication strategies based on humor and storytelling can overcome the influence of sociodemographic determinants [65,85], as emotional engagement helps sustain attention and improves message retention [86]. However, some concerns arise regarding these results due to sampling and dropout bias, which are common in online experiments and may introduce systematic differences between participants who complete the study and those who withdraw prematurely.

The evidence regarding cultural moderators remains complex. The influence of religiosity at 2-month follow-up on the effectiveness of debunking messages is consistent with the “Worldview Backfire Effect” model, according to which deeply rooted spiritual and cultural beliefs tend to prevail even when confronted with contradictory information [67,87]. Conversely, prebunking messages—designed to present scientific facts and anticipate misinformation to build resistance against false claims—are not influenced by levels of spirituality, representing valuable tools for public health communication [88]. Likewise, the significant preference for vaccine information content featuring individuals from the same ethnic background [55,63]—consistent with social and ethnic identification theories—needs to be considered in communication strategies [89]. These findings should be interpreted with caution, as they are primarily based on self-reported outcomes.

However, despite the strength of randomized designs in establishing causality, this body of evidence is characterized by substantial heterogeneity in interventions (ranging from internet memes to community radio) and outcome measures (hypothetical intention vs validated medical records). Moreover, restricting inclusion to studies with a sample size greater than 1000 potentially introduces selection bias, leading to the omission of valuable evidence from smaller experimental studies.

### Strengths and Limitations

To the best of our knowledge, this review is among the first comprehensive examinations of media-based vaccine communication strategies aimed at enhancing vaccine adherence. It is methodologically robust, drawing on a very large evidence base and integrating both traditional and digital media influences on vaccination. It advances a precision-focused communication model shaped by key sociodemographic factors, and its strict adherence to PRISMA, PROSPERO, and multiple quality-assessment tools ensures a transparent and reliable synthesis.

Nevertheless, several limitations should be acknowledged. First, many studies assess vaccination intentions rather than actual behaviors and often exclude booster doses, which are critical for successful immunization programs. Second, the predominance of cross-sectional designs restricts causal inference. Experimental studies are relatively few, comprising only 8 out of the 36 [60-67] included studies. RCTs generally showed a low risk of bias in randomization and intervention delivery, supporting the evidence that certain communication strategies can positively influence vaccination attitudes, although confounding and self-reported outcomes require cautious interpretation. Third, data collected from specific online platforms introduce selection bias due to participants’ distinct sociodemographic and technological profiles. Fourth, publication bias may have occurred, as studies reporting significant communication effects are more likely to be published. To mitigate this risk, strict inclusion criteria and comprehensive quality assessments were applied. Finally, although this review included a broad set of relevant studies, some pertinent works may still have been missed.

### Conclusions

In conclusion, by synthesizing large-scale, fragmented evidence on the correlation between media and vaccine acceptance, and highlighting the role of sociodemographic determinants, this review moves beyond isolated analyses, offering a systematic and comprehensive examination of how multichannel media strategies can be leveraged to mitigate vaccine hesitancy and bolster adherence.

This work departs from previous studies in its dual focus on both traditional and digital media through the lens of sociodemographic moderation. By identifying that tailored communication—particularly through ethnic identification, storytelling, and prebunking—can overcome deeply rooted barriers, it provides a critical framework for future public health interventions.

To advance the field, future research priorities must shift toward rigorous longitudinal and experimental designs—particularly in low-income settings—and the standardized evaluation of emerging tools to validate causal links between communication and behavioral change.

The results of this review may provide starting points for orienting communication strategies toward a “precision” model. In this sense, profiling sociodemographic factors may be applied in designing strategies tailored to recipients’ characteristics and cultural contexts.

## Appendix

Multimedia Appendix 1 PRISMA (Preferred Reporting Items for Systematic Reviews and Meta-Analyses) 2020 checklist.

Multimedia Appendix 2 Full search strategy.

## Abbreviations

M-MERSQI: Modified Medical Education Research Study Quality Instrument
PICO: Population, Intervention/Exposure, Comparison, and Outcome
PRISMA: Preferred Reporting Items for Systematic Reviews and Meta-Analyses
PROSPERO: International Prospective Register of Systematic Reviews
RCT: randomized controlled trial
RoB: Risk-of-Bias 2.0 tool for randomized trials
ROBINS: Risk of Bias in Non-randomized Studies of Interventions
WHO: World Health Organization

## Data Availability

A template reporting the extracted data may be available upon motivated request and approval by the research team.
