# Supplementary material for: Media Exposure and Its Association With Vaccine Attitudes, Intentions, and Hesitancy: Systematic Review
Source: J Med Internet Res. 2026 Apr 28;28:e74280. doi: 10.2196/74280 (PMC13168859; doi:10.2196/74280)
Supplement: Multimedia Appendix 2 [file jmir_v28i1e74280_app2.docx]

**Full search strategy**

PubMed

( "Vaccination"[Mesh] OR "Immunization"[Mesh] OR vaccin*[tiab] OR immuniz*[tiab] OR immunis*[tiab] ) AND ( "Vaccine Hesitancy"[Mesh] OR "Health Knowledge, Attitudes, Practice"[Mesh] OR "Patient Acceptance of Health Care"[Mesh] OR "Health Literacy"[Mesh] OR hesitan*[tiab] OR accept*[tiab] OR uptake[tiab] OR refus*[tiab] OR confidence[tiab] OR trust*[tiab] OR literacy[tiab] OR "parent* concern*"[tiab] OR "risk perception*"[tiab] OR KAP[tiab] ) AND ( "Health Communication"[Mesh] OR communication[tiab] OR "risk communication"[tiab] OR message*[tiab] OR campaign*[tiab] ) AND ( "Artificial Intelligence"[Mesh] OR "Machine Learning"[Mesh] OR "Natural Language Processing"[Mesh] OR chatbot*[tiab] OR "large language model*"[tiab] OR LLM*[tiab] OR algorithm*[tiab] OR "Mass Media"[Mesh] OR "Social Media"[Mesh] OR "Internet"[Mesh] OR "Television"[Mesh] OR "Newspapers"[Mesh] OR "Advertising as Topic"[Mesh] OR "social media"[tiab] OR internet[tiab] OR online[tiab] OR web[tiab] OR television[tiab] OR TV[tiab] OR newspaper*[tiab] OR advertising[tiab] OR marketing[tiab] OR media[tiab] OR Facebook[tiab] OR Twitter[tiab] OR X[tiab] OR YouTube[tiab] OR TikTok[tiab] OR Instagram[tiab] OR misinformation[tiab] OR disinformation[tiab] OR infodemic*[tiab] )

Scopus

TITLE-ABS-KEY( (vaccin* OR immuni?ation*) AND ( "vaccine hesitan*" OR hesitan* OR accept* OR uptake OR refus* OR confidence OR trust* OR literacy OR "parent* concern*" OR (knowledge W/3 attitude*) OR KAP ) AND ( communication OR "risk communication" OR message* OR campaign* ) AND ( "artificial intelligence" OR "machine learning" OR "natural language processing" OR chatbot* OR "large language model*" OR algorithm* OR "social media" OR "mass media" OR internet OR television OR tv OR newspaper* OR advertising OR marketing OR media OR Facebook OR Twitter OR X OR YouTube OR TikTok OR Instagram OR misinformation OR disinformation OR infodemic* ) )

Web of Science

TS=( (vaccin* OR immuni?ation*) AND ( communication OR "risk communication" OR literacy OR hesitan* OR accept* OR uptake OR refus* OR confidence OR trust* OR "parent* concern*" OR (knowledge NEAR/3 attitude*) OR KAP ) AND ( "artificial intelligence" OR "machine learning" OR "natural language processing" OR chatbot* OR "large language model*" OR algorithm* OR "social media" OR "mass media" OR internet OR television OR tv OR newspaper* OR advertising OR marketing OR media OR Facebook OR Twitter OR X OR YouTube OR TikTok OR Instagram OR misinformation OR disinformation OR infodemic* ) )
